# The mediating effect of ICU nurses’ teamwork level between workload and missed nursing care in infection prevention and control

**DOI:** 10.3389/fmed.2026.1930402

**Published:** 2026-10-29

**Authors:** Fukai Yang, Zixin Wang, Yajuan Zhang, Li Zhang, Wenjie Zhou, Wenqi Tang

**Affiliations:** Department of Nursing, Shanghai Sixth People's Hospital Affiliated to Shanghai Jiao Tong University School of Medicine, Shanghai, China

**Keywords:** ICU nurse, IPC-related omission, mediating effect, teamwork, workload

## Abstract

**Objective:**

To explore the mediating effect of ICU nurses’ teamwork level between workload and Missed Nursing Care in Infection Prevention and Control (MNCIPC) and to provide references for improving the status of MNCIPC in ICUs and enhancing the quality of clinical nursing.

**Method:**

A total of 350 ICU nurses from six tertiary hospitals in Shanghai were recruited from January to March 2025 via convenience sampling. This study utilized a general data questionnaire, the MNCIPC Survey, the Nurse–Nurse Collaboration Scale (NNCS), and the NASA Task Load Index (NASA-TLX). Structural equation modeling (SEM) was employed to investigate the mediating effect of ICU nurses’ teamwork level on the relationship between workload and MNCIPC.

**Results:**

A total of 328 ICU nurses ultimately participated in the survey. Workload was positively correlated with MNCIPC (*p* < 0.01) and negatively correlated with nurses’ teamwork level (*p* < 0.01). Nurses’ teamwork level was negatively correlated with MNCIPC (*p* < 0.01). The mediating effect analysis revealed that nurses’ teamwork level partially mediated the relationship between workload and MNCIPC, with an indirect effect value of 0.055, accounting for 21.74% of the total effect based on bootstrapping with 5,000 samples.

**Conclusion:**

The level of teamwork among ICU nurses may be a mediating variable between workload and MNCIPC. Nursing managers may closely monitor ICU nurses’ workload and actively foster a work environment that promotes mutual support and close collaboration to enhance teamwork, thereby improving the current situation of MNCIPC.

## 1 Introduction

Healthcare-associated infections (HAIs) pose a significant global public health challenge, especially in intensive care unit (ICU) settings. A meta-analysis (1) revealed that the worldwide incidence of HAIs is 14%, increasing at an annual rate of 6%. Data from the National Medical Institution Infection Surveillance System of China indicated that the prevalence of HAIs in Chinese hospitals in 2022 was 1.64%, with rates ranging from 0.78% to 2.19% across hospitals of different sizes (2). ICU patients are at higher risk of infection due to severe illness, frequent invasive procedures, and weakened immune systems. A survey (3) showed that the incidence of HAIs among ICU patients could be as high as 35%. HAIs led to longer hospital stays, increased mortality risk, higher healthcare costs (4–6), and poorer clinical outcomes. ICU nurses play a vital role in preventing and managing HAIs (7), and their infection control practices directly influence HAI rates (8, 9). Missed nursing care, first proposed by Kalisch in 2006, refers to the phenomenon where necessary nursing activities are partially or completely omitted or delayed in clinical practice for various reasons (10). Research (11–13) indicates that HAI-related omissions are positively correlated with the incidence of HAIs. Building on Kalisch's work, and through a literature review and qualitative interviews with infection prevention and control (IPC) experts, Henderson et al. (14) developed the MNCIPC Scale to assess MNCIPC in Australia. An in-depth exploration of the factors and mechanisms influencing MNCIPC in ICUs holds significant clinical value. This study not only provides a scientific basis for creating targeted intervention strategies but also helps reduce HAI rates, ensure patient safety, improve healthcare quality, and lessen the healthcare burden.

Nursing workload refers to the total services provided by nurses to patients, including physical effort, emotional fluctuations, time, and energy spent during the nursing process (15, 16). ICU nurses spend most of their time providing direct care to critically ill patients and therefore face a significantly higher workload compared to nurses in other departments (17), which greatly affects the effectiveness of infection control measures. A scoping review (18) identified workload as a key factor influencing MNCIPC. An excessive nursing workload raises the risk of Healthcare-Associated Infections (HAIs) (19). An observational study (20) revealed a negative relationship between workload and nurses’ adherence to hand hygiene, resulting in inadequate implementation of IPC measures due to high work pressure and limited time. Additionally, a prospective study (21) found that excessive workload increases the risk of HAIs in ICU patients. However, the specific mechanisms connecting ICU nurses’ workload to MNCIPC behaviors remain insufficiently understood.

Nurse teamwork refers to the process by which nursing team members effectively communicate, collaborate, and support each other to accomplish patient care tasks. Research (22) indicates that poor communication and collaboration are linked to patient safety incidents (e.g., adverse events and medical errors). Effective teamwork and communication are essential for ensuring the quality and safety of patient care. Given the rapidly changing conditions and frequent emergencies typical of ICU patients, efficient task delegation and close collaboration within the nursing team are crucial (23). Existing research suggests that the level of nurse teamwork affects the occurrence of missed care. A study in Turkey (24) identified nurse teamwork as a significant predictor of missed nursing care. Multiple studies (25–28) further suggested a negative correlation between nurse teamwork levels and missed care. A cross-sectional survey involving 1162 ICU nurses (29) found that nurses reporting higher satisfaction with teamwork also reported fewer instances of missed care. Moreover, teamwork has been significantly associated with HAI risk. Studies show that poor collaboration among ward staff greatly increases patients’ risk of acquiring HAIs (30). A US study across 44 neonatal ICUs (31) demonstrated that a positive teamwork climate was negatively correlated with HAI rates in very low birth weight infants. Similarly, an Italian study (32) found that higher team effectiveness among ICU staff was linked to lower HAI incidence. However, research specifically examining the relationship between ICU nurse teamwork levels and missed IPC care is currently lacking.

The Job Demands-Resources (JD-R) model is a well-known theoretical framework in occupational health psychology and organizational behavior, used to examine how the work environment impacts employee behavior and well-being. According to the JD-R model, elements of work conditions or the work environment can be classified as either job demands or job resources (33). Job demands include physical, social, or organizational aspects of the job that require ongoing physical or psychological effort and are linked to physiological or psychological costs, such as workload, role conflict, and emotional demands (34). Job resources refer to aspects of the job that help achieve work goals, lessen job demands, and promote personal growth and development, including social support, autonomy, and rewards (35). This model is widely used to identify factors that influence employee well-being, engagement, and various individual and organizational outcomes (36). In nursing, the JD-R model has been applied to explore nurse burnout (37), work engagement (35), turnover intention (38), and job satisfaction (39), among other indicators. As a job demand, research (40) shows that ICU nurses’ workload is a significant factor influencing missed care, with a positive correlation established between nurse workload and missed care (41). As a job resource, previous studies (41–43) indicate that nurse teamwork not only improves job satisfaction (42) but also promotes patient-centered care (44). Furthermore, research (17, 26) shows a significant negative correlation between ICU nurses’ teamwork levels and missed care, suggesting that teamwork may buffer the negative effects of workload.

Guided by the JD-R model, this study aims to examine how ICU nurses’ workload (job demand) and teamwork level (job resource) affect MNCIPC, analyzing the intrinsic relationship among these variables to improve ICU nursing management.

The study proposes the following theoretical hypotheses, as shown in Figure 1.

Hypothesis 1: Workload and nurses’ teamwork level directly affect MNCIPC.
Hypothesis 2: Nurses’ teamwork level mediates the relationship between workload and MNCIPC.

**Figure 1:**
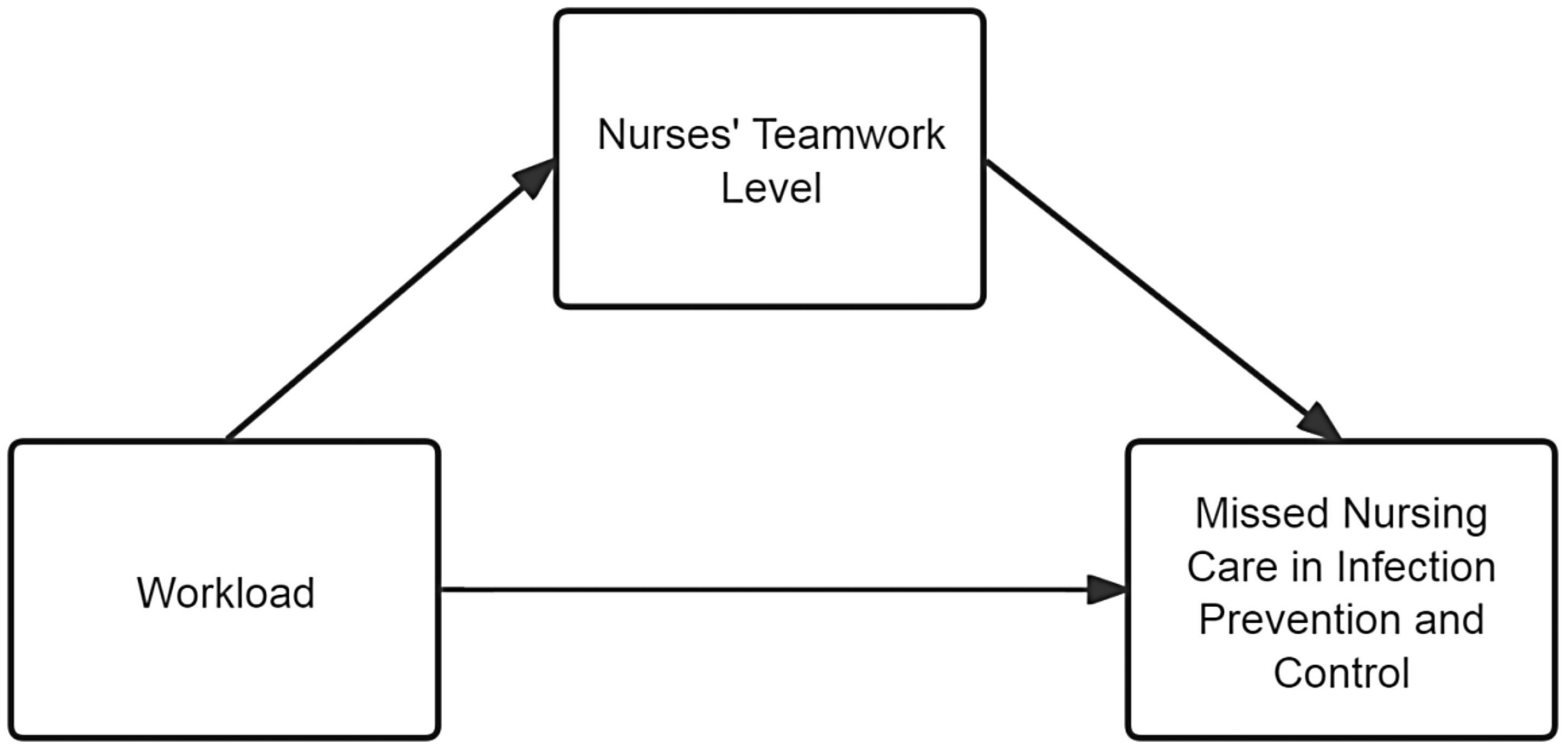
Theoretical hypotheses.

## 2 Methods

### 2.1 Aim of the study

The primary objective is to examine how ICU nurses’ workload (job demand) and teamwork level (job resource) affect MNCIPC. The secondary objective is to examine whether nurse teamwork mediates the relationship between workload and MNCIPC.

### 2.2 Participants and sample size

This study used a convenience sampling method. Participants were recruited from ICU nurses working in six tertiary Grade-A hospitals in Shanghai between January and March 2025. These hospitals have established collaborative relationships with the research team, making it easier to access participants. While convenience sampling is highly practical, it may affect how well the sample represents the broader population and can limit how widely the findings can be applied. To reduce potential bias, we set strict inclusion criteria and enrolled a sufficiently large sample size to improve the robustness and reliability of the results.

The inclusion criteria were as follows: ① had a valid nursing practice license; ② were currently working in an ICU; ③ had at least one year of ICU work experience; and ④ provided informed consent and voluntary participation. The exclusion criteria were as follows: ① Nurses were absent from duty for various reasons; and ② Nurses underwent rotation or training.

Based on Kendall's sample size estimation method (45), the sample size should be 5–10 times the number of variables. This study involved 18 variables. The final sample size was 216, determined based on 10 times the number of variables, and accounted for invalid questionnaires with a 20% margin of error. A total of 350 questionnaires were distributed, and 328 valid questionnaires were returned, resulting in an effective response rate of 93.71%.

This study was approved by the Ethics Committee of Shanghai First People's Hospital [Approval No. Yuan Lun Kuai (2024) 276]. All participants volunteered and provided full informed consent.

### 2.3 Measurements

#### 2.3.1 General data questionnaire

The general data questionnaire included personal information (gender, age, marital status, and education level) and work-related information (employment type, professional title, years of ICU experience, ICU type, average monthly income, number of patients assigned during the last shift, job satisfaction, and turnover intention).

#### 2.3.2 MNCIPC care survey (MNCIPC)

The MNCIPC survey was used to evaluate the level of MNCIPC. This scale was developed by Australian researchers Henderson et al. (14) and subsequently translated into Chinese by Lv et al. (46). The Chinese version comprises Subscale B (31 items, types and frequency of MNCIPC activities) and Subscale C (20 items, reasons for MNCIPC). Subscale B uses a 5-point Likert scale (1 = Never Missed, 2 = Rarely, 3 = Occasionally, 4 = Often, 5 = Always), with higher scores indicating more frequent omissions of specific nursing activities. Subscale C employs a 4-point scale (1 = Not a reason, 2 = Minor reason, 3 = Moderate reason, 4 = Major reason), where higher scores indicate that the factor is a more significant reason for missed nursing care. The overall Cronbach's alpha coefficients for the subscales were 0.973 and 0.902, with test-retest reliability coefficients of 0.917 and 0.957, respectively, and content validity indices of 0.973 and 0.949. Li et al. (47) used this scale to assess MNCIPC levels among nurses in tertiary Grade A hospitals in China, demonstrating internal consistency with a Cronbach's*α* of 0.941. This study used Subscale B to assess MNCIPC, which had a Cronbach's *α* of 0.965.

#### 2.3.3 NASA task load index (NASA-TLX)

The NASA-TLX scale was used to measure participants’ workload. Developed by Hart et al. (48) and translated into Chinese by Liang et al. (49), this scale has been utilized to assess nurses’ workload (50–53). The Chinese version of the NASA-TLX consists of two dimensions: load perception and self-evaluation. Load perception encompasses four items (mental demand, physical demand, temporal demand, and performance), while self-evaluation comprises two items (effort and frustration). A 20-point line represents each item, scored from 0 to 20. The total workload score is the sum of the six items, ranging from 0 to 120, with higher scores indicating greater workload. Its validity and reliability were suggested in various studies involving ICU nurses in China (54–56). In this study, the Cronbach's *α* coefficient was 0.786.

#### 2.3.4 Nurse–nurse collaboration scale (NNCS)

The NNCS was utilized to measure nurses’ teamwork level. This scale was developed by Dougherty et al. (57) and translated into Chinese by Shi (58) in 2016 for assessing nurses’ teamwork level. The Chinese version of the NNCS comprises 25 items distributed across four dimensions: conflict resolution (8 items), communication (4 items), professionalism (7 items), and the shared process (6 items). The scale employs a 4-point Likert scale (Strongly Disagree = 1, Disagree = 2, Agree = 3, Strongly Agree = 4). The total score varies from 25 to 100, with higher scores reflecting better collaboration among nurses. Its Cronbach's *α* coefficient was 0.934, split-half reliability was 0.880, and test-retest reliability was 0.837. The validity and reliability were confirmed in various studies of ICU and emergency nurses in China (59, 60). In this study, the Cronbach's *α* coefficient was 0.964.

### 2.4 Common method bias test

To assess potential common method bias (CMB), this study employed Harman's single-factor test and compared a confirmatory factor analysis (CFA) single-factor model with the theoretical multi-factor model. First, we subjected all 62 items from the three scales to an unrotated exploratory factor analysis. The results showed that nine factors had eigenvalues greater than 1, and the first principal factor accounted for only 31.77% of the total variance, well below the conventional 50% threshold. This indicated no strong evidence that a single common factor explained most of the measurement variance (61, 62). Furthermore, CFA compared the theoretical multi-factor model with a single-factor model to examine the potential impact of common method bias. The results showed that the theoretical multi-factor model fit significantly better than the single-factor model, in which all items were loaded onto a single latent variable. Specifically, the changes in fit indices of the single-factor model relative to the theoretical multi-factor model were *Δ*CFI = −0.296, *Δ*TLI = −0.306, *Δ*RMSEA = 0.038, and *Δ*SRMR = 0.086, indicating that attributing all items to a single latent variable could not adequately explain the covariance structure among items. Thus, from a CFA model-comparison perspective, the data also did not show clear evidence of a single common factor dominating the measurement relationships (61). In summary, neither Harman's single-factor test nor the CFA single-factor model comparison revealed obvious signs of common method bias.

### 2.5 Data collection

Data collection was completed electronically via a Chinese online questionnaire platform (Wenjuanxing). After obtaining consent from the nursing departments of the participating hospitals, the researchers contacted and trained the head nurses of each department. They explained the research purpose, content, significance, and instructions for completion in detail, emphasizing the anonymity and confidentiality of the questionnaire while clarifying the inclusion and exclusion criteria. The head nurses subsequently distributed the questionnaire to eligible ICU nurses, who completed it anonymously based on informed consent.

To ensure result quality, the researchers implemented management measures on the Wenjuanxing platform: only one submission was permitted per device or IP address, and all questions had to be answered before finalizing the submission. After data collection, two researchers performed quality checks and removed questionnaires deemed invalid. The criteria for invalid questionnaires included (a) obvious patterned responses, (b) contradictory answers, and (c) very short completion times.

### 2.6 Data analysis

The data were exported from Wenjuanxing to SPSS 27.0 software and carefully checked by two researchers. Descriptive analyses of personal and work-related information were conducted using frequencies and percentages. The analysis of MNCIPC, workload, and nurses’ teamwork levels utilized the mean and standard deviation (SD). T tests and analysis of variance (ANOVA) were employed to examine the impact of demographic factors on MNCIPC. Additionally, multiple linear regression and Pearson correlation analyses were performed to assess the relationships among MNCIPC, workload, and nurses’ teamwork levels. Finally, AMOS 26.0 software was used to investigate the path relationships among these three variables via a mediating model. Model fit was evaluated using *χ*^2^/df ratio, Goodness-of-Fit Index (GFI) (GFI > 0.9), Adjusted Goodness-of-Fit Index (AGFI), Comparative Fit Index (CFI), Normed Fit Index (NFI), and Root Mean Square Error of Approximation (RMSEA) to determine the reasonableness of the model. Furthermore, *p* < 0.05 was considered statistically significant, and the bootstrapping method with 5,000 replicated samples was used to calculate multiple mediating effects and bias-corrected confidence intervals (CI).

## 3 Results

### 3.1 Participants’ demographic characteristics

A total of 328 questionnaires were collected. Descriptive analysis of the participants’ basic characteristics revealed that most nurses in the sample were female (82.62%), were aged between 25 and 35 years (59.45%), and that 57.93% were married. In terms of education, 84.45% had a bachelor's degree or higher. The most common professional title was Nurse Practitioner (53.66%). Most nurses worked in general ICUs (61.59%), and 86.28% reported no intention to leave. See Table 1.

**Table 1:** Demographic characteristics of participants and scores of MNCIPC (*n* = 328).

Variables	Category	N(%)	MNCIPC（SD）	t/f	*p*
Gender				3.241	0.001*
	Male	57 (17.38)	54.70 ± 21.21		
	Female	271 (82.62)	47.44 ± 13.85		
Age				1.298	0.274
	≤25	43 (13.11)	49.33 ± 14.25		
	25–35	195 (59.45)	49.61 ± 17.06		
	＞35	90 (27.44)	46.46 ± 12.47		
Marriage Status				0.027	0.978
	Married	190 (57.93)	48.73 ± 17.67		
	Unmarried/Others	138 (42.07)	48.68 ± 13.94		
Education Level				1.682	0.188
	Secondary or below	51 (15.55)	46.96 ± 14.34		
	Bachelor's	271 (82.62)	48.80 ± 15.87		
	Master's or above	6 (1.83)	59.17 ± 8.70		
Employment Type				1.21	0.306
	Permanent Staff	124 (37.81)	50.46 ± 14.29		
	Agency Staff	13 (3.96)	49.46 ± 23.54		
	Contract Staff	178 (54.27)	47.80 ± 16.06		
	Other	13 (3.96)	43.54 ± 10.00		
Professional Title				0.321	0.810
	Staff Nurse	44 (13.41)	49.82 ± 12.86		
	Nurse Practitioner	176 (53.66)	48.82 ± 17.43		
	Nurse Supervisor	103 (31.41)	48.31 ± 13.58		
	Associate/Chief Nurse	5 (1.52)	43.00 ± 6.78		
Years in ICU				6.2	<0.001*
	1 to 5	116 (35.37)	51.47 ± 15.09		
	5 to 10	96 (29.27)	50.72 ± 17.75		
	10 to 15	65 (19.82)	46.52 ± 15.10		
	≥15	51 (15.54)	41.39 ± 9.44		
ICU Type				15.545	<0.001*
	Internal ICU	13 (3.96)	40.23 ± 8.87		
	Surgical ICU	54 (16.46)	56.31 ± 18.09		
	Emergency ICU	51 (15.55)	59.08 ± 20.40		
	General ICU	202 (61.59)	44.78 ± 11.29		
	Other	8 (2.44)	44.12 ± 16.68		
Average Monthly Income				5.09	0.002*
	0–5000	14 (4.27)	38.93 ± 5.23		
	5000–10000	143 (43.60)	46.57 ± 14.25		
	10000–15000	138 (42.07)	50.49 ± 16.01		
	≥15000	33 (10.06)	54.61 ± 19.04		
No. of Patients Last Shift				5.565	<0.001*
	1	14 (4.27)	41.29 ± 7.27		
	2	45 (13.72)	42.04 ± 11.31		
	3	130 (39.63)	48.13 ± 14.19		
	4	67 (20.43)	49.87 ± 15.27		
	≥5	72 (21.95)	54.26 ± 19.39		
Job Satisfaction				9.896	<0.001*
	Dissatisfied	6 (1.83)	73.33 ± 19.91		
	Neutral	114 (34.76)	52.18 ± 15.56		
	Satisfied	161 (49.08)	46.67 ± 14.70		
	Very Satisfied	47 (14.33)	44.11 ± 13.93		
Turnover Intention				−3.707	<0.001*
	No	283 (86.28)	47.46 ± 13.68		
	Yes	45(13.72)	56.56 ± 23.13		

* *p* < 0.01.

### 3.2 MNCIPC, workload, and nurses’ teamwork level

The average score for MNCIPC among the 328 participants was 48.70 ± 15.59. The average workload score was 86.53 ± 18.18, with sub-dimension scores of load perception (63.91 ± 12.88) and self-evaluation (22.62 ± 9.62). The average score for nurses’ teamwork level was 77.82 ± 11.28, with sub-dimension scores including Conflict Resolution (22.95 ± 3.81), Communication (12.77 ± 2.17), Professionalism (22.45 ± 3.48), and Shared Process (19.73 ± 3.17). Additionally, higher levels of MNCIPC (*p* < 0.05) were reported by male nurses, those with 1-5 years of ICU experience, individuals working in emergency ICUs, those managing five or more patients during their last shift, those with an average monthly income exceeding 15,000 CNY, those dissatisfied with their jobs, and those planning to leave. See Table 1.

### 3.3 Correlations among MNCIPC, workload, and ICU nurses’ teamwork level

The results showed that a higher workload was associated with increased levels of MNCIPC (r = 0.329, *p* < 0.01). A higher level of teamwork among nurses was linked to fewer MNCIPC instances (r = −0.328, *p* < 0.01). Additionally, as workload increased, teamwork among nurses decreased (r = −0.307, *p* < 0.01).

### 3.4 Multiple linear regression

Multiple linear regression was used to examine how variables identified as statistically significant in the univariate analysis impact MNCIPC see Table 2.

**Table 2:** Multiple linear regression analysis of factors influencing MNCIPC.

Variable	Unstandardized Coefficient (B)	Standard Error (SE)	Standardized Coefficient (*β*)	t	*P*	Tolerance	VIF
Constant	58.018	7.464		7.773	<0.001		
Gender	−4.977	2.016	−0.121	−2.469	0.014*	0.930	1.075
Years in ICU	−2.362	0.723	−0.163	−3.267	0.001*	0.903	1.107
ICU Type（Ref：Internal ICU）							
Surgical ICU	11.798	4.193	0.281	2.814	0.005*	0.324	3.086
Emergency ICU	11.14	4.348	0.259	2.562	0.011*	0.319	3.135
General ICU	2.744	3.832	0.086	0.716	0.474	0.256	3.906
Other ICU	3.617	6.018	0.036	0.601	0.548	0.630	1.588
Average Monthly Income	3.122	1.072	0.146	2.911	0.004*	0.890	1.124
No. of Patients Last Shift	1.42	0.747	0.1	1.901	0.058	0.801	1.248
Job Satisfaction	−4.289	1.088	−0.196	−3.942	<0.001*	0.906	1.104
Turnover Intention	5.103	2.234	0.113	2.284	0.023*	0.919	1.089

R^2^ = 0.290; F = 12.959.

* *p*＜0.05.

### 3.5 Mediating effect of ICU nurses’ teamwork level on the relationship between workload and MNCIPC

Structural equation modeling (SEM) was employed to investigate further the relationships among MNCIPC, workload, and ICU nurses’ teamwork levels. Non-significant variables were removed from the model. Subsequently, model modifications were performed to examine the relationships between: ICU work experience (years) and job satisfaction, ICU work experience (years) and Emergency ICU type, ICU work experience (years) and Surgical ICU. The model was illustrated in Figure 2.

**Figure 2:**
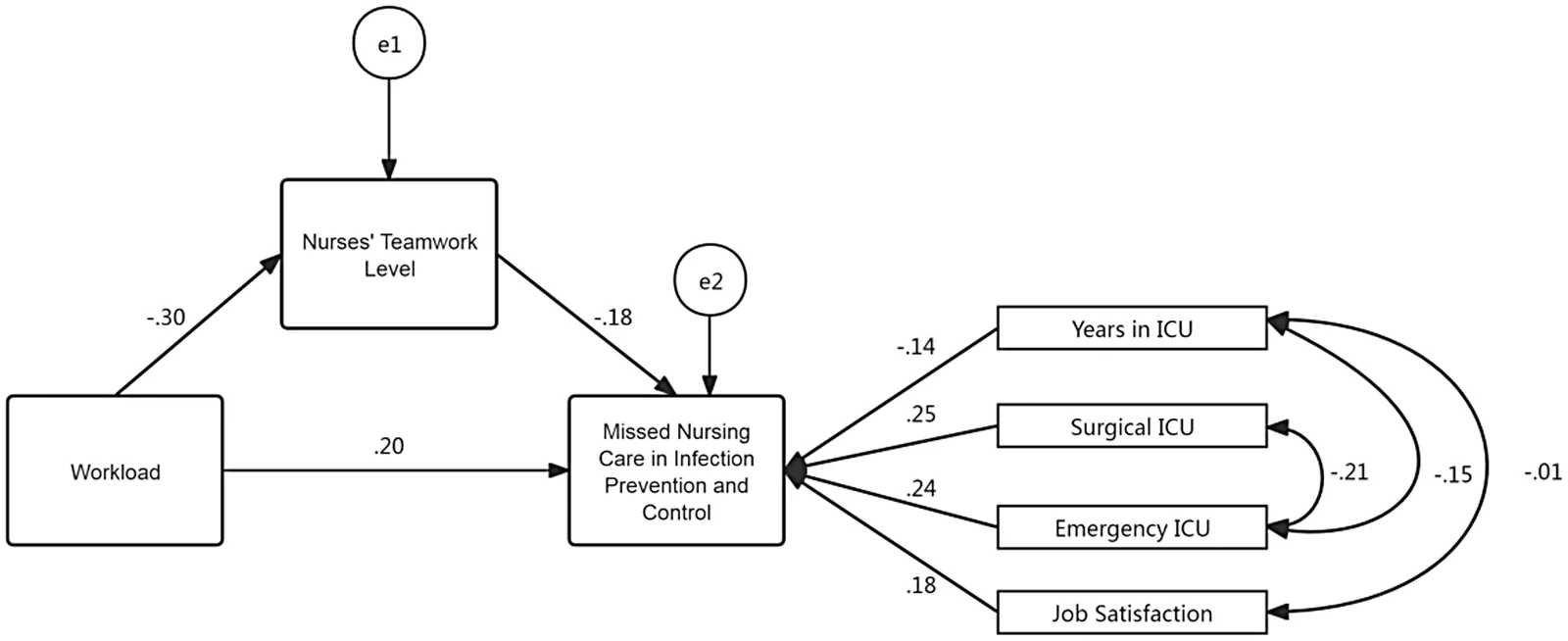
Mediating effect model of nurses’ teamwork level between workload and MNCIPC.

The mediating role of ICU nurses’ teamwork level was evaluated using 5,000 bootstrap samples and 95% confidence intervals. All fit indices were satisfactory see Table 3. Additionally, workload had a significant direct effect on MNCIPC (*β* = 0.198, *p* < 0.001). The teamwork level of ICU nurses also significantly affected MNCIPC (*β* = −0.182, *p* < 0.001). These results support Hypothesis 1. Moreover, the indirect mediating effect of workload → nurses’ teamwork level → MNCIPC was 0.055 (*p* < 0.001), representing 21.74% of the total effect. Therefore, the findings suggest that nurses’ teamwork level partially mediates the relationship between workload and MNCIPC, supporting Hypothesis 2. Refer to Tables 4, 5 and Figure 2.

**Table 3:** Final model fit criteria and indices.

Fit Index	*χ*^2^/df	SRMR	GFI	AGFI	NFI	CFI	RMSEA
Standard	<5	<0.05	>0.90	>0.90	>0.90	>0.90	<0.08
Result	1.53	0.046	0.991	0.963	0.95	0.981	0.040

**Table 4:** Effect of nurses’ teamwork level on the relationship between workload and MNCIPC.

Path	Unstandardized Coefficient	Standardized Coefficient	Standard Error	t	*p*
Workload→Nurses’ Teamwork Level	−0.187	−0.301	0.032	−5.750	<0.001
Nurses’ Teamwork Level→Missed Nursing Care in IPC	−0.248	−0.182	0.068	−3.665	<0.001
Workload→Missed Nursing Care in IPC	0.167	0.198	0.042	3.980	<0.001

**Table 5:** Mediating effect analysis of nurses’ teamwork level between workload and missed IPC care.

Effect Type	Effect	SE	Bootstrapping 95% CI	
			Lower	Upper
Direct Effect	0.198	0.042	0.107	0.300
Mediating Effect	0.055	0.020	0.023	0.102
Total Effect	0.253	0.046	0.165	0.346

## 4 Discussion

The results of this study showed that the mean score for MNCIPC among ICU nurses was 48.70 ± 15.59, which is below the midpoint of the total scale score (63.00), indicating a moderate-to-low level of MNCIPC in ICUs. This finding aligns with the level reported among nurses in tertiary hospitals but is slightly lower than the value reported by Li et al. (47) (48.94 ± 15.06). The study also found that sex, years of ICU experience, ICU type, average monthly income, job satisfaction, and turnover intention all affected the level of MNCIPC. Specifically, male nurses reported higher levels of MNCIPC than female nurses, which agrees with previous research (63). This difference may be related to cultural norms, knowledge levels, education, and training. In many cultures, women are often associated with domestic cleanliness and hygiene (64). Gender differences in cultural norms may cause female nurses to focus more on IPC, while male nurses, who may lack similar social expectations, tend to follow lower IPC standards. Additionally, men are traditionally seen as having advantages in physical strength and technical skills. Therefore, male nurses might be more involved in tasks requiring higher technical proficiency or physical exertion (65), which could limit their ability to handle essential IPC tasks simultaneously. Knowledge level is also a key factor; studies show that male nurses have significantly lower hand hygiene knowledge compared to females (64), and ICU male nurses scored lower on tests about preventing central line-associated bloodstream infections (66). Furthermore, insufficient education and training play important roles. Research (67) indicates that female healthcare workers report receiving more hand hygiene training than their male counterparts. The frequency of training is an important factor in nurses’ adherence to standard precautions (68). However, these explanations are speculative, and the impact of gender on missed nursing care in infection prevention and control still warrants further investigation.

This study also revealed that junior ICU nurses had higher levels of MNCIPC, which aligns with the findings of previous studies (69–71). The ICU environment is highly stressful and intense. Junior nurses, who are not yet accustomed to this fast-paced setting, may find it challenging to carry out IPC measures within limited time frames effectively. Additionally, they might have received less IPC training and lack sufficient knowledge of the latest IPC guidelines. Moreover, their limited experience may cause them to undervalue the importance of IPC measures. Nurses’ IPC knowledge and beliefs may be associated with their adherence to IPC behaviors (14, 72–74).

This study suggested that the type of ICU may influence nurses’ missed nursing care at IPC levels, with emergency ICUs showing the highest rates, similar to previous findings (75, 76). Patients in emergency or ICUs often have complex, rapidly changing conditions that require frequent resuscitation and urgent interventions. This leads to very high work intensity, which can easily result in omissions in IPC care. Prior research (77, 78) suggested that urgent patient conditions and unexpected increases in patient numbers are major reasons for missed care. Additionally, emergency or ICU nurses often face conflicts when deciding between following IPC protocols and addressing urgent care needs. When IPC measures (e.g., hand hygiene, wearing PPE) cause delays in resuscitation, nurses tend to prioritize immediate, life-saving actions, which can reduce IPC compliance. Research (79) has shown poor hand hygiene adherence among healthcare workers during trauma resuscitation. Furthermore, overcrowded environments during emergencies hinder nurses’ ability to perform IPC measures (80).

Furthermore, the average monthly income may influence the level of MNCIPC, which aligns with the findings of Yang et al. (29). Among ICU nurses, those with higher average incomes also reported higher MNCIPC levels, possibly because they undertook more demanding clinical tasks or management responsibilities. This study showed that job satisfaction may also affect MNCIPC levels, similar to previous findings (81–84). Nurses’ job satisfaction has a negative correlation with HAI incidence (85). Leaving intention was another factor influencing MNCIPC, consistent with earlier research (86). Nurses intending to leave are less engaged in their work and tend to overlook IPC details.

This study revealed that ICU nurses’ workload may have a significant direct effect on MNCIPC. This finding aligns with previous research results (41, 87). As a department with a high level of work intensity in the hospital, ICU nurses’ workload primarily comes from two factors: the need to respond to sudden changes in patients’ conditions at any time and the requirement to complete multiple complex nursing procedures within a limited time. This persistent high-pressure work environment impacts the implementation of IPC measures through various mechanisms. From a time perspective, an overloaded workload causes nurses to experience “time poverty.” Chang et al. (88) reported that nurses’ hand hygiene compliance significantly decreases under high workloads. Research (14) indicates that when nurses handle multiple urgent tasks simultaneously, they prioritize interventions that directly threaten life and neglect the importance of IPC measures. From a physiological standpoint, sustained high-intensity work leads to obvious signs of physical fatigue. In this exhausted state, nurses are more likely to forget hand hygiene steps, resulting in reduced compliance (19, 89). Psychological and emotional factors are equally crucial. A long-term high workload triggers symptoms of occupational burnout, such as emotional exhaustion (90). Research (91) shows that burnout is negatively associated with nurses’ hand hygiene compliance. This psychological influence is often subtle, and nurses may not fully recognize changes in their IPC behaviors. Therefore, nursing managers could scientifically assess the workload and intensity of ICU nurses, adjusting staffing dynamically based on patient acuity and census to meet clinical demands. This may help to lessen workload, ensuring nurses have adequate time and energy to implement IPC measures and reducing missed care. At the same time, specialized training for junior nurses may be increased, indirectly decreasing their workload by enhancing competency. Additionally, managers could monitor nurses’ psychological well-being, foster a supportive team environment, establish psychological support systems, and regularly offer mindfulness-based stress reduction, group counseling, and peer support programs. These strategies may reduce psychological stress, lower burnout, and improve IPC behaviors and quality.

This study found that nurse teamwork levels may influence MNCIPC: higher teamwork may correlate with fewer MNCIPC incidents, consistent with previous research (17, 26). A study (30) associates poor staff collaboration with HAI occurrence. Effective teamwork reduces MNCIPC through several mechanisms: first, it enhances workload sharing. Teamwork decreases individual nurse burdens (27). In supportive teams, nurses share responsibilities, support one another, and proactively assist with tasks, resulting in fewer instances of missed nursing care (17, 92). Second, it promotes efficient information exchange. Good teamwork encourages sharing and communication among nurses (93, 94), ensuring critical information is conveyed quickly and thereby reducing delays or omissions that lead to missed care. Additionally, teamwork increases nurse efficiency, allowing timely delivery of care measures (95). Therefore, healthcare institutions could develop a comprehensive system to promote nurse teamwork, including interventions at three levels: 1) Capability Building: Regularly conduct Team Strategies and Tools to Enhance Performance and Patient Safety (TeamSTEPPS) training and simulations to improve team communication and collaboration skills, ultimately reducing missed IPC care. 2) Management Mechanisms: Hold daily structured team handover meetings using the SBAR (Situation-Background-Assessment-Recommendation) communication tool to ensure accurate transfer of critical patient information, decreasing missed care caused by poor communication. 3) Environmental Support: Establish a dedicated incentive fund for nursing team collaboration to foster a cooperative work environment, strengthen team cohesion, improve overall team performance, promote timely implementation of IPC measures, and prevent delays or omissions.

This study found a positive correlation between workload and missed IPC care (*β* = 0.198, *p* < 0.001), and a negative correlation between nurse teamwork levels and missed IPC care (*β* = −0.182, *p* < 0.001). The mediating effect of teamwork between workload and missed IPC care was 0.055, accounting for 21.74% of the total effect. This indicates that workload may directly influence MNCIPC and may have an indirect effect through teamwork. Specifically, ICU nurses’ IPC behaviors are affected by workload, and changes in workload impact team dynamics, which in turn influence MNCIPC. When workload is manageable, nurses have more time and energy, promoting greater willingness to share information and coordinate tasks, thereby strengthening teamwork. The approach to IPC shifts from “individual responsibility” to “team collaboration.” Conversely, excessive workload causes physical and mental fatigue, reducing the willingness to communicate and collaborate. In such situations, nurses tend to focus solely on their tasks, paying less attention to colleagues and team needs (96), making IPC adherence more reliant on individual effort. Additionally, teamwork mediated the relationship between workload and MNCIPC, ultimately affecting ICU IPC quality. Even under high workload, strong team collaboration can help reduce missed IPC care. Therefore, nursing managers could recognize the link between workload, teamwork, and MNCIPC, understand the negative impact of high workload on IPC quality, and ensure adequate staffing in ICUs.

This study shows that the mediating effect of nurses’ teamwork level between workload and missed nursing care in IPC is moderate. This suggests that improving teamwork may help mitigate the partial negative impact of high workload on missed IPC care. In nursing environments where human resources are scarce and difficult to replenish promptly, enhancing teamwork can be a feasible adjunctive strategy to reduce missed IPC care. Nursing managers could monitor ICU nurses’ workload and alleviate it by optimizing human resource allocation, adjusting shift schedules, and introducing auxiliary nursing staff, thereby potentially reducing missed IPC care and improving IPC quality.

## 5 Limitations

This study has the following limitations. First, the sampling method used convenience sampling and recruited ICU nurses solely from tertiary Grade-A hospitals in Shanghai. It excluded nurses from secondary hospitals, specialized hospitals, and private healthcare institutions, and did not include nurses from regions with varying levels of economic development. Convenience sampling inherently cannot ensure the sample's representativeness relative to the population, and the homogeneity of the sample source further limits the generalizability of the study's conclusions. Given significant differences in healthcare resource allocation and nursing management practices across regions, these findings require further verification for generalizability. Regarding nursing human resource allocation, the nurse-to-bed ratio in Shanghai ICUs is higher than in most provinces and regions across China (97). In regions with strained human resources, nurse teamwork may serve as a more critical “buffering mechanism,” with mutual support and information sharing among colleagues playing a more prominent role in maintaining IPC practices. Consequently, the proportion of the mediating effect may be higher than 21.74%. Second, this study assessed missed IPC care using self-report, which may introduce reporting bias compared with direct observation. Future studies should adopt probability sampling strategies and expand the sample to include healthcare institutions of different levels and types across different regions. Third, this study did not collect data on shift type (day vs. night), which may confound missed nursing care. Future studies should include shift type as a covariate. Additionally, although Harman's single-factor test and CFA comparison did not indicate significant common method bias, self-report measures may still be subject to residual social desirability bias. Finally, mixed-methods designs incorporating direct observation should be used to obtain objective data on missed IPC care among ICU nurses.

## 6 Conclusion

This study suggests that ICU nurses’ workload may not only directly affect missed IPC care but also indirectly influence it through the mediating role of nurse teamwork levels. This suggests that nursing managers may enhance teamwork levels and reduce missed IPC care by implementing effective measures to alleviate nurses’ workload. This study was conducted solely in tertiary Grade-A hospitals in Shanghai, and the sample's representativeness and generalizability are subject to certain limitations. The conclusions still require validation in broader populations. Future research should investigate the workload, teamwork levels, and missed IPC care of ICU nurses across different regions, hospital levels, and types of healthcare institutions nationwide, thereby providing further evidence to improve IPC care.

## Data Availability

The raw data supporting the conclusions of this article will be made available by the authors, without undue reservation.
